# Mixed-phase enabled high-rate copper niobate anodes for lithium-ion batteries†

**DOI:** 10.1039/d4ta07548j

**Published:** 2025-01-08

**Authors:** B. Maarten Jager, Luuk Kortekaas, Johan E. ten Elshof, Jan-Willem G. Bos, Moniek Tromp, Mark Huijben

**Affiliations:** a Zernike Institute for Advanced Materials, University of Groningen 9747 AG Groningen Netherlands moniek.tromp@rug.nl; b MESA+ Institute for Nanotechnology, University of Twente 7500 AE Enschede Netherlands m.huijben@utwente.nl; c EaStCHEM School of Chemistry, University of St Andrews KY16 9ST St Andrews UK j.w.g.bos@st-andrews.ac.uk

## Abstract

The advancement of rapid-response grid energy storage systems and the widespread adoption of electric vehicles are significantly hindered by the charging times and energy densities associated with current lithium-ion battery technology. In state-of-the-art lithium-ion batteries, graphite is employed as the standard negative electrode material. However, graphite suffers from polarization and deteriorating side-reactions at the high currents needed for fast charging. Transition metal-oxide anodes are attractive alternatives due to their enhanced power density. However, often these anodes make use of toxic or scarce elements, significantly limiting their future potential. In this work, we propose a new, facile solid-state synthesis method to obtain non-toxic, abundant, mixed-phase copper niobate (Cu_*x*_Nb_*y*_O_*z*_) anodes for lithium-ion batteries. The material consists of various phases working synergistically to deliver high electrochemical capacities at exceptional cycling rates (167 mA h g^−1^ at 1C, 95 mA h g^−1^ at 10C, 65 mA h g^−1^ at 60C and 37 mA h g^−1^ at 250C), large pseudocapacitive response (up to 90%), and high Li^+^ diffusion coefficient (1.8 × 10^−12^ cm^2^ s^−1^), at a stable capacity retention (99.98%) between cycles. Compared to graphite, at a comparable energy density (470 W h L^−1^), the composite material exhibits a 70 times higher power density (27 000 W L^−1^). These results provide a new perspective on the role of non-toxic and abundant elements for realizing ultrafast anode materials for future energy storage devices.

## Abstract 2

Mixed-phase copper niobate anodes for lithium-ion batteries consisting of various phases work synergistically to deliver high electrochemical capacities at exceptional cycling rates.

## Graphical abstract

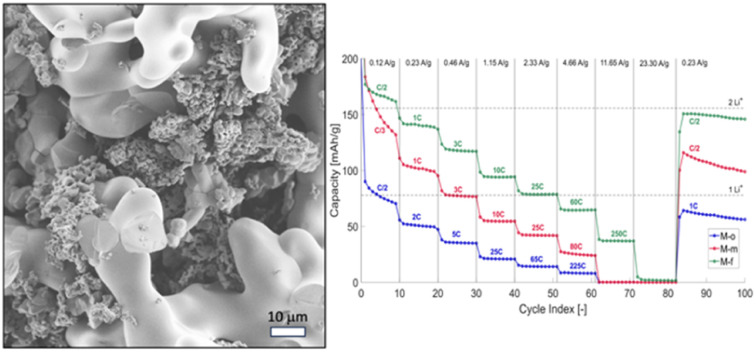

## 1 Introduction

In 2024, fossil fuels remain the world's primary energy source, as over 60% of the shared global energy supply is derived from coal, oil and natural gas.^1^ To limit greenhouse gas emissions and reduce global warming, it is necessary to continuously develop renewable energy sources and expand their global energy share. One of the main issues of solar-, wind- and hydropower is their large dependency on local weather conditions, leading to major fluctuations in energy generation.^2^ To prevent electric energy shortages, an expansive energy grid is crucial for balancing local shortages with surpluses from another region. Additionally, excesses in generated energy must be temporarily stored in stationary energy storage facilities, to be readily discharged during periods of energy shortage. However, to phase out fossil fuels entirely, a highly responsive grid is essential, as larger areas require higher current densities to manage peak demand and supply. Therefore, fast charging and discharging storage systems are critical for ensuring grid responsiveness and stability.^2^ Furthermore, the surplus of electric energy is mostly curtailed by the deactivation of green energy generators, due to inadequate energy storage infrastructure.^3–5^ This highlights the need for the continued development of novel battery energy storage systems capable of efficiently charging and discharging as required by local or regional electricity demands. Batteries are interesting candidates for energy storage due to their high energy density, operational flexibility, and rechargeability.^6^ Apart from the domestic energy grid, the US Department of Energy has identified the fast charging of electric vehicles (EVs) as a critical challenge to be overcome, in order for the mass adoption of EVs over combustion engine vehicles.^7–9^ This will result in a reduction of greenhouse gas emissions and greater national energy securities, due to the lessened degree of fossil dependence, further emphasizing the need for fast charging battery technology.^10^

At present, the market share of rechargeable batteries is dominated by lithium-ion batteries (LIBs) and, owing to their superior energy density and long reusability, continues to grow annually.^11,12^ Commercial LIBs typically make use of graphite anodes, wherein lithium-ions intercalate reversibly during operation. Fully lithiated, graphite has a theoretical capacity of 339 mA h g^−1^ at a 0.2 V potential *versus* Li/Li^+^ and a modest 10% volume expansion. This results in cycle lifetimes of up to several thousands, at a fair capacity retention (up to 85% after 1000 cycles).^11,13^ However, the low operating voltage limits the charging rate, due to the risk of lithium metal plating and lithium dendrite growth.^11,14^ Furthermore, current graphite anodes suffer from slow Li^+^-intercalation, due to sluggish kinetics.^15,16^ When subjected to high currents, graphite anodes undergo polarizations and deteriorating side-reactions, resulting in lower intercalation capacities.^15^ Lastly, safety concerns of graphite exist due to the combination of its flammable carbon, with the use of typically flammable electrolyte and possible oxygen release during cathodic cycling.^14^ Hence, in order to realize the full potential of LIBs, in both stationary energy storage as well as EVs, anode materials capable of operating at high (dis)charge rates must be developed with stable, highly reversible capacities.

Transition metal-oxide anodes are attractive alternatives to graphite in LIBs due to their enhanced power density in combination with their high energy density and safe operation.^17^ Nickel is widely used in both anodes and cathodes, with batteries consuming 3–4% of all nickel globally.^18^ Due to an anticipated annual growth of 39% in the battery nickel industry, nickel shortages may loom on the horizon.^18,19^ Next to this, nickel mining has been linked to poor working conditions and high environmental impact.^18–20^ In contrast, copper is a more energy-efficient alternative, with greater overall production and abundance.^18,19,21^ Therefore, a rise in the usage of copper over nickel will result in a broader distribution of material usage, enhancing flexibility and lowering the costs of production. Another upcoming compound in LIB anodes is niobium oxide. This material has been studied for its unique ‘room-and-pillar’ framework structure, which provides a host lattice for fast lithium-ion intercalation.^22^ Niobium(v) oxide (Nb_2_O_5_) phases have been shown to possess high reversible capacities (150 mA h g^−1^) at moderate charge rates (1C), which rapidly degrade upon increase of the applied current density.^23^

Combination of a divalent 3d metal-oxide (MO) with niobium(v) oxide, forms a columbite (MNb_2_O_6_) crystal structure with a high working potential similar to Li_4_Ti_5_O_12_ (LTO).^24^ Columbite materials are known for their exceptionally stable layered structure, which permits the reversible intercalation of lithium ions. Theoretically, this structure can accommodate up to three lithium ions per unit cell while preserving its layered host–guest configuration with extensive one-dimensional channels. This capability ensures high stability and capacity, making columbite materials promising alternatives to graphite anodes in LIBs.^25^ Current work on columbite anode materials in literature featured Co,^26^ Fe,^27^ Mn,^28^ Zn,^28^ and Cu,^25^ of which overviews are shown in Tables S1 and S2 in the ESI.† Currently, the highest reversible capacity at fast discharge is determined for NiNb_2_O_6_, as obtained by Xia *et al.*,^29^ at 250 mA h g^−1^ at 1C and 100 mA h g^−1^ at 10C. Recent work^30–32^ on copper niobate anodes focused on steering away from the columbite crystal structure, towards a shear ReO_3_-type niobate structure. This crystal orientation enhances Li^+^-diffusion, allowing for increased cycling rates, but significantly lowers the overall Cu^2+^ concentration (down to 1.5 at%). Generally in copper niobate materials, the Cu^2+^-ions (t_2g_^6^e_g_^3^) are responsible for most of the electronic conductivity, due to their 9 free 3d electrons,^32^ compared to no free 4d electrons for Nb^5+^ (t_2g_^0^e_g_^0^). Hence, in literature,^25,30^ a trade-off has been observed between conductivity (higher Cu^2+^ ratio) and electrochemical capacity (higher Nb^5+^ ratio). Furthermore, stability and electrolyte compatibility issues have been reported in ReO_3_-type anodes.^30^ To overcome the limited reversible capacity of columbite-type CuNb_2_O_6_ and the limited electronic conductivity of ReO_3_-type copper niobate anodes, a material has to be designed that incorporates the advantages of both materials.

Here, we present the first study on mixed-phase copper niobate anodes based on composite materials with enhanced electrochemical stability and electronic conductivity, exhibiting highly reversible capacities at exceptional cycling rates. The anodes consist of various phases working synergistically, where the copper-rich phases provide electrons needed for conductivity and stability, while the niobium-rich phases are responsible for the redox activity, and hence the reversible capacity. A facile solid-state synthesis method is used to obtain both pure monoclinic and orthorhombic phases of CuNb_2_O_6_ and provide new insights in their electrochemical nature. Additionally, by slightly increasing the molar ratio of Nb over Cu, a composite material is formed consisting of several phases. These mixed-phase anode compounds are completely composed of abundant and non-toxic elements and are suitable for fast and efficient energy storage.

## 2 Materials and methods

### 2.1 Powder synthesis

To form the pure copper niobate powder, CuO (>99%, Boom B.V., the Netherlands) and t-Nb_2_O_5_ (99.9%, Sigma-Aldrich) or h-Nb_2_O_5_ (annealing of t-Nb_2_O_5_ at 975 °C, 3 h) were weighed off in a 1 : 1 molar ratio and mixed for 24 hours at 200 rotations per minute (rpm) in 10 mL ethanol. Afterwards, the dispersion was transferred to a platinum crucible, and the ethanol was left to evaporate at a hot plate set at 120 °C for 1 hour. Then, the crucible was placed in a Nabertherm RHTH 120/300/16 tube oven, set at a heating and cooling rate of 5 °C min^−1^, under ambient atmosphere. The monoclinic phase was produced at 700 °C for 12 hours, the orthorhombic phase at 1100 °C for 4 hours (Table 1).

**Table 1:** Synthesis methods used to form the phase-pure monoclinic (P-m) and orthorhombic (P-o) copper niobate powders, as well as the performance-optimized monoclinic (M-m), orthorhombic (M-o) and biphase (M-f) anode powders. For M-f a molar excess of 5% Nb was used to steer the synthesis towards the formation of new phases

Sample	Target phase	Nb	Molar ratio Cu : Nb [—]	Time at *T*_ma*x*_ [h]	*T*_ma*x*_ [°C]
P-m	Monoclinic	t-Nb_2_O_5_	1 : 1	12	700
P-o	Orthorhombic	t-Nb_2_O_5_	1 : 1	4	1100
M-m	Monoclinic	h-Nb_2_O_5_	1 : 1	8	700
M-o	Orthorhombic	t-Nb_2_O_5_	1 : 1	24	1000
M-f	Biphase	h-Nb_2_O_5_	1 : 1.05	8	700

### 2.2 Battery cell assembly

After the annealing step, 56 mg copper niobate powder was mixed with 16 mg carbon black in a mortar and pestle for 15 minutes. The powder mixture was transferred to a 5 mL plastic Eppendorf tube. Subsequently, 8 mg of polyvinylidene fluoride (PVDF), suspended in 160 μL *N*-methyl-2-pyrrolidone (NMP) was pipetted into the tube. The mixture was ultrasonicated for 30 minutes until a thick slurry was produced, that was spread out over a copper foil, to form a thin and homogeneous layer, with a concentration of active material of 1–3 mg cm^−2^. The hot plate was set to 70 °C for 3 hours to evaporate the solvents. The electrode, together with clean, empty electrochemical cells was dried under a vacuum at 60 °C overnight, before assembly in a glove box under an argon atmosphere.

The circular (1 cm^2^) copper niobate electrodes were tested in a half-cell against pure lithium, where the used electrolyte was 400 μL 1.0 M LiPF_6_ (Sigma-Aldrich, battery grade), in a 1 : 1 volume ratio ethylene carbonate/dimethyl carbonate (EC/DMC), combined with a 1 mm thick glass fiber separator (ECC1-01-0012-B/L, EL-Cell).

### 2.3 Characterization techniques

To characterize the precursor powder and resultant electrodes; CV, GDC, nitrogen physisorption, SEM, XANES, XPS, XRD, and XRF were used.

The electrochemical measurements (CV, GDC, and GITT) were performed on a multichannel potentiostat (VMP-300, BioLogic) with the EC-Lab software, at room temperature. The measured potentials were reported against Li^+^/Li. The occurring electrochemical reactions were gauged by cyclic voltammetry, with a working voltage set between 1 and 3 V *vs.* Li^+^/Li, at a scan rate of 0.1 mV s^−1^, which was repeated twice. Galvanostatic charge/discharge measurements were performed at 1C (233 mA g^−1^), at a working voltage set between 1 and 3 V *vs.* Li^+^/Li, to determine the specific capacity of the anodes, and the battery stability.

Nitrogen physisorption (BET) measurements were conducted to determine the specific surface area of the produced powders, using a Gemini VII Micromeritics device. Before analysis, approximately 1 gram of sample powder was first degassed by a N_2_ flow at 100 °C for 1 hour and subsequently at 300 °C for 3 hours. BET experiments were conducted at 77.3 K suspended in liquid nitrogen, using N_2_ as analysis gas and an evacuation rate of 350 mm_Hg_ min^−1^. Specific surface areas were calculated *via* the BET method.

Scanning electron microscopy was performed in a Zeiss Merlin HR-SEM at room temperature, at a potential of 1.40 kV and a working distance of 2.5 mm. Images were taken with the energy-selective backscattered electron detector, the InLens immersion primary electron detector, and the high-efficiency secondary electron detector, to visualize the micro- and nanostructure of the material.

Cu K-edge (8978.9 eV) and Nb K-edge (18 985.6 eV) X-ray absorption near edge structure spectra were collected *via* transmission mode at the Balder beamline^33^ of the MAX IV Laboratory synchrotron light source in Lund, Sweden. The liquid N_2_-cooled double crystal monochromator equipped with Si(111) crystals was calibrated to the first inflection of Se-foil (12 658 eV). The X-ray beam at the sample position was defocused to be used in a spot size of 200 × 400 μm^2^. Double-shielded split-collector gas-filled ionization chambers, one before the sample (*I*_0_: 20% absorption level, 500 mbar N_2_ + 500 mbar He for Cu XANES, and 400 mbar Ar^+^ 600 mbar N_2_ for Nb XANES) and one after the sample (*I*_1_: 60% absorption level, 60 mbar Ar^+^ 1000 mbar N_2_ for Cu XANES and 100 mbar Kr + 1000 mbar N_2_ for Nb XANES) were used as detectors for the incoming X-ray intensity and transmission. The optimal sample mass was calculated by the CatMass^34^ software and combined with a 2× excess of polyethylene powder to be pressed into pellets for measurement. The measurements were conducted at ambient temperature and were repeated 3 times. The X-ray absorption spectra were energy calibrated by recording of XANES spectra of copper and niobium foils, where for copper the first inflection and for niobium the second inflection was set as (*E*_0_). The unknown oxidation states of the samples were determined *via* linear fitting of the formal valency of the sample powders to specified measured samples. The experimental absorption spectra were treated by standard procedures for analysis, using the software program package Demeter.^35^

X-ray photoelectron spectroscopy was performed to study the oxidation states of copper, niobium, and oxygen, using an Omicron Nanotechnology GmbH surface analysis system, with a photon energy of 1486.7 eV with an Al Kα X-ray source. The emission current was set to 12.5 mA and the anode to 15 kV.

X-ray diffraction experiments were used to study the synthesized powders. Measurements were taken using a Malvern PANalytical X'Pert PRO diffractometer system, equipped with a powder-measurement stage. The diffractor used Cu Kα radiation, a 10 mm mask, 1/16° slit, at a step size of 0.0033° and 70.1 s per step. Rietveld refinement analyses were conducted on the prepared materials using the Profex software program.^36^

X-ray fluorescence spectroscopy was performed to determine the elemental ratio of Cu, Nb, and O in the pristine CuNb_2_O_6_ powder. XRF was done in a Bruker S8 Tiger WDXRF, equipped with a Rh X-ray tube.

## 3 Results & discussion

### 3.1 Phase synthesis

**Fig. 1:**
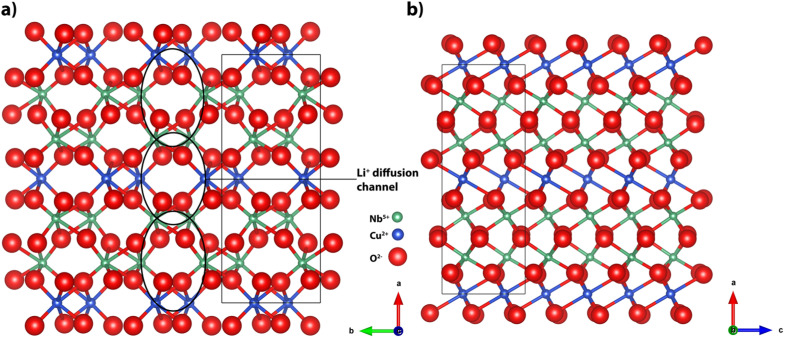
(a) Front view and (b) side view of the orthorhombic CuNb_2_O_6_ columbite crystal structure. Elements are shown with their ionic radii, the Li^+^-diffusion channels are only open in the 〈0 2 1〉 vector, and fully closed along the 〈0 0 1〉 vector.

Copper niobate crystallizes in the columbite phase,^37^ exhibiting either a monoclinic^28,37^ (*P*12_1_/*c*1) or an orthorhombic^25,37^ (*Pbcn*) crystal structure, depending on the synthesis temperature. The monoclinic system exhibits lattice parameters of 14.173, 5.762 and 5.006 Å, with a *γ* angle of 91.67°.^38^ The orthorhombic system is slightly smaller, with 14.097, 5.613 and 5.123 Å lattice parameters.^38^ The oxygen-ions form corner-shared octahedra around the Cu^2+^- and Nb^5+^-ions, resulting in zig-zagging Cu^2+^-ion chains. Per unit cell, three Li^+^-ions can intercalate into the circular channels of 2.88 Å in diameter,^29,38^ resulting in a theoretical capacity of 233 mA h g^−1^ for Li_3_CuNb_2_O_6_. This lithiation occurs by partial reduction of the transition metal ions, Cu^2+^ and Nb^5+^, during discharging of the half-cell. According to Xia *et al.*^29^ and Li *et al.*,^25^ both transition metal ions contribute to the lithium intercalation process. Niobium ions partially reduce from Nb^5+^ to Nb^4+^ and even from Nb^4+^ to Nb^3+^, while copper ions are reduced from Cu^2+^ to Cu^+^. Even reduction to Cu^0^ could possibly be achieved, analogous to the Ni^0^ detected by X-ray photoelectron spectroscopy (XPS) after cycling of NiNb_2_O_6_ electrodes.^29^Fig. 1 shows the crystal structure and lattice directions of orthorhombic CuNb_2_O_6_. It is clearly visible that the Li^+^-ions can only diffuse through the structure in a single direction along the 〈0 2 1〉 vector before being placed between four surrounding octahedrons. The open columbite crystal structure combines fast ion intercalation and high energy storage capacity, which are desired electrode properties for enhanced LIBs.

**Fig. 2:**
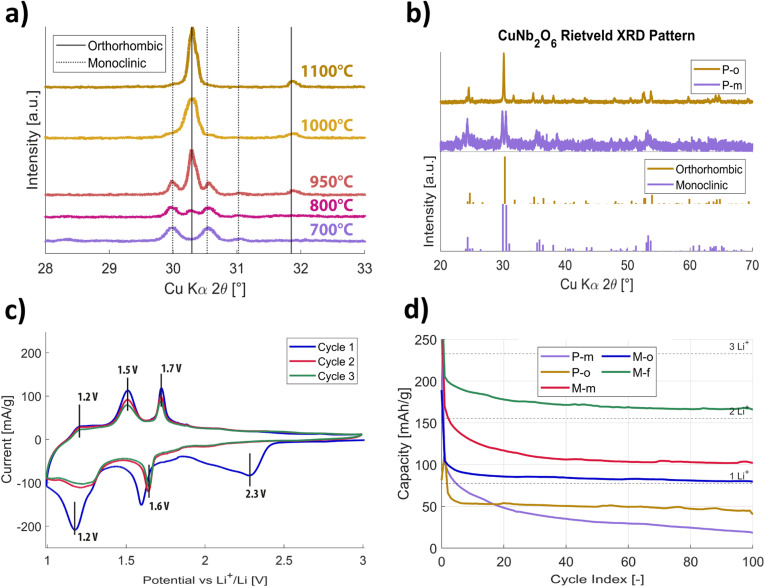
(a) X-ray diffractograms (XRD) showing the annealing temperature dependence of the CuNb_2_O_6_ phases, vertical lines indicate compound reference peaks.^38,41^ (b) X-ray diffractograms of phase-pure (P-m and P-o) CuNb_2_O_6_ powder samples, with XRD diffractograms of the crystal phases as ref. 38 and 41. (c) Electrochemical characterization of phase-pure monoclinic CuNb_2_O_6_ (P-m) *via* cyclic voltammetry, measured in the potential range from 3.0 to 1.0 V (*vs.* Li/Li^+^), at a scanning rate of 0.1 mV s^−1^. (d) GDC graphs, obtained for the phase-pure monoclinic and orthorhombic CuNb_2_O_6_ electrodes (P-m & P-o), compared to the performance-optimized derivatives (M-m, M-o & M-f), cycled at a current density of 233 mA g^−1^ (1C).

The monoclinic structure is known to be formed at temperatures above 650 °C, and the structure transforms into the orthorhombic structure from 900 °C.^39,40^ To obtain phase-pure copper niobate samples, the annealing temperatures were varied between 700 and 1100 °C (Fig. 2a). The diffraction peaks of the sample annealed at 700 °C match the monoclinic phase, while for 1100 °C the peaks indicate the presence of a fully orthorhombic phase.^38,41^ Detailed Rietveld analysis confirmed the synthesis of phase-pure monoclinic (further referred to as P-m) and orthorhombic (referred to as P-o) CuNb_2_O_6_ in both cases (Fig. 2b). Both powders exhibited sharp peaks, indicating high crystallinity and purity of the samples. For temperatures between 800 and 1000 °C, the XRD data indicate the presence of both monoclinic and orthorhombic phases.

### 3.2 Electrochemical characterization

The copper niobate electrodes were subjected to cyclic voltammetry (CV) (Fig. 2c and S1†) in a half-cell setup against a lithium metal counter-electrode, whilst using 1.0 M LiPF_6_ in ethylene carbonate/dimethyl carbonate (EC/DMC) as electrolyte. The electrodes were cycled between 3.0 and 1.0 V *vs.* Li^+^/Li at a scan rate of 0.1 mV s^−1^. The reductive (bottom) part of the cycle corresponds to the discharging of the electrochemical cell, as Li^+^ intercalates in the copper niobate electrode, requiring reduction of the local transition metal ions, while the upper part of the voltammogram corresponds to the cell's charge, under oxidative conditions. Looking at the cyclic voltammogram, one can clearly see a discrepancy in the electrochemical behaviour between the first and subsequent cycles. In the reductive part, the broad peak around 2.3 V disappears in later cycles. This indicates that a redox reaction takes place in the first cycle, changing the chemical environment in the half-cell irreversibly. This reaction is likely related to the formation of a solid electrolyte interface (SEI) layer on the electrode's surface.

Around 2.3 V, the CuNb_2_O_6_ reacts irreversibly with lithium-ions and is decomposed into LiNb_3_O_8_, NbO_2_ and Li_2_O, initiating the growth of the SEI layer *via* Li_2_O:^42^

1 CuNb_2_O_6_ + 5Li^+^ + 5e^−^ → LiNb_3_O_8_ + NbO_2_ + 2Li_2_O + 2Cu

The second major peak can be seen at 1.6 V. Here, the propagation of the SEI layer formation slightly affects the position and intensity of the main peak.^43,44^ At 1.2 V, another large peak is seen to disappear over the first cycle, presumably due to the presence of CuO impurities or unreacted copper niobate particles, reacting with lithium-ions to form copper(i) oxide, lithium oxide, and niobium(v) oxide:^45,46^

2 CuO + *x*Li^+^ + *x*e^−^ → Li_*x*_CuO

or

3 2CuNb_2_O_6_ + 2Li^+^ + 2e^−^ → Cu_2_O + 2Nb_2_O_5_ + Li_2_O

*Via* these reactions, a large fraction of the copper ions is irreversibly reduced, creating a SEI layer, while forming Nb_2_O_5_. In subsequent cycles, a number of reversible chemical reactions occur, starting with the partial reduction of Nb(v) to Nb(iv), in the newly created LiNb_3_O_8_ phase at 1.6/1.7 V^42,47,48^

4 LiNb_3_O_8_ + *x*Li^+^ + *x*e^−^ ↔ Li_1+*x*_Nb_3_O_8_

At 1.3/1.5 V and 1.1/1.2 V, the formed niobium(iv) oxide reversibly intercalates lithium under partial reduction to Nb^3+^:^49,50^

5 NbO_2_ + *y*Li^+^ + *y*e^−^ ↔ Li_*y*_NbO_2_

Two different peaks are observed for the same niobium oxidation state, as a result of the different chemical environment of the reducing atoms. At 1.2 V, in the reductive part of the cycle, a reversible, broad peak can be seen. This is associated with the structural destruction and subsequent formation of Cu_2_O:^45,51^

6 2CuO + 2Li^+^ + 2e^−^ → Cu_2_O + Li_2_O or 2Li_*x*_CuO → Cu_2_O + *x*Li_2_O

here, Cu(ii) oxide is reduced to Cu(i) oxide, under the formation of an amorphous layer of Li_2_O, following the growth of the solid electrolyte interface (SEI). The formation of the SEI layer limits the electrochemical activity of the electrode. In subsequent cycles, at 1.2/2.3 V partial re-oxidation of Cu_2_O into Li_*x*_CuO occurs, resulting in a small amount of working capacity:^45,46,51^

7 2Li_*x*_CuO ↔ Cu_2_O + *x*Li_2_O

The repeated structural destruction and transformation of Li_*x*_CuO into CuO and amorphous Li_2_O progresses the formation of the SEI layer, hindering Li^+^-diffusion to the copper niobate particles, leading to poor cycleability and rate performance.^46^

Galvanostatic discharge/charge (GDC) cycling of phase-pure samples of orthorhombic (P-o) or monoclinic (P-m) CuNb_2_O_6_, over 100 cycles at a current density of 233 mA g^−1^ (1C), matches the observations made by CV (Fig. 2d and S2†). The first P-o and P-m discharge cycles show a significant irreversible capacity loss. For P-o, in subsequent cycles, the reversible capacity decreases until the fourth cycle, to 80 mA h g^−1^ (*versus* 233 mA h g^−1^ theoretical capacity). In search for better capacity retention, synthesis conditions used to produce the phase-pure orthorhombic (o) and monoclinic (m) CuNb_2_O_6_ powders were changed (Table 1). The approach was chosen to steer away from obtaining phase-pure CuNb_2_O_6_, by changing the reaction time and temperature in an effort to move towards ReO_3_-type anode materials, with enhanced reversible capacity.^30,32^ Moreover, the high-temperature crystallized phase of niobium(v)oxide (h-Nb_2_O_5_) by annealing of t-Nb_2_O_5_ at 975 °C for 3 h was used, which was previously shown^47^ to enhance battery performance due to its open crystal structure. This led to the formation of impure mixed-phase monoclinic (referred to as M-m) and orthorhombic (referred to as M-o) powders, as well as a biphase, containing both the columbite copper niobate orthorhombic and monoclinic phases (referred to as M-f), with superior electrochemical performances.

**Fig. 3:**
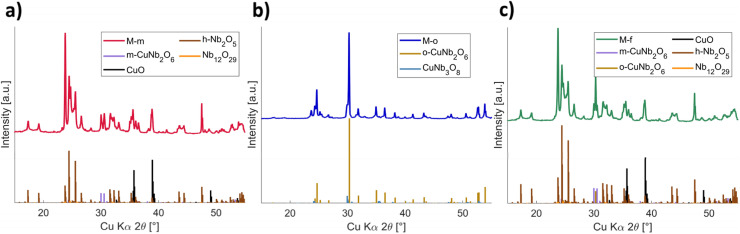
XRD phase analyses of the (a) M-m, (b) M-o and (c) M-f compounds by splitting of the detected peaks in the XRD diffractogram into individual peaks for the multiple phases present in the samples.

For synthesis of the biphase (M-f) compound, an excess of 5 mol% of Nb with respect to Cu was used in an effort to increase the material's electrochemical capacity, in the same approach as the ReO_3_-type copper niobate anodes contain a lowered the Cu^2+^ concentration. This resulted in an apparent formation of both the monoclinic and the orthorhombic CuNb_2_O_6_ phases, next to several others (Fig. 3).

Comparison of the GDC electrochemical data of the performance-optimized mixed-phases M-o, M-m and M-f compounds to the phase-pure P-o and P-m compounds over the first 100 cycles, shows that the mixed-phases stabilize at much higher capacities, with superior lithium storage and coulombic efficiencies (Fig. 2d and Table 2). This indicates that the formation of multiple phases has a profound effect on the achieved reversible capacity and stability. Specifically, the M-f compound shows a high lithium-ion storage capability, and thus electrochemical capacity, at exceptional coulombic efficiency and capacity retention.

**Table 2:** Comparison of the electrochemical properties of P-m, P-o, M-o, M-m and M-f compounds. Here *C*_p,rev_ yields the electrochemical capacity after 100 cycles at 1C, while CE and RT_Cp_ indicate the cell's averaged coulombic efficiency, and the capacity retention between cycles, as calculated between the 2nd and the 100th cycles

Property	P-m	P-o	M-o	M-m	M-f
*C*_p,rev_ at *j* = 233 mA g^−1^ (1C, 100 cycles) [mA h g^−1^]	25	45	80	102	167
Li^+^ per unit cell	0.32	0.58	1.03	1.31	2.15
Percentage of *C*_p,theo_ [%]	10.72	19.31	34.33	43.78	71.67
CE [%]	106.01	112.49	100.85	100.57	100.98
RT_Cp_ [%]	98.37	99.51	99.57	99.77	99.81

### 3.3 Structural characterization

X-ray fluorescence (XRF) data of the phase-pure CuNb_2_O_6_ compounds P-m and P-o (Table S3†), show that the compounds contain atomic concentrations theoretically expected for copper niobate. The mixed-phase samples however, have very different values, with an overall decreased concentration of copper atoms. This presumably leads to a distortion of the crystal lattice and the formation of new crystal phases, affecting the Li^+^-diffusivity and consequently the electrochemical capacity, as well as the material's stability. Fig. 3 shows X-ray diffractograms of the M-m, M-o and M-f compounds. Compared to the diffractograms of the phase-pure samples (Fig. 2b), many new peaks can be identified, indicating the formation of new phases next to the monoclinic (P-m) and orthorhombic (P-o) phases, see also Fig. S3.† The XRD peaks of the columbite monoclinic and orthorhombic copper niobate were used to calculate the lattice parameters and crystallite sizes of the P-m, P-o, M-o and M-m compounds (Table S4†), *via* Rietveld refinement (Fig. S4†) in the software program Profex.^36^ P-m and P-o match with the theoretical data (Table S1†). The M-o, M-m and M-f compounds show larger deformations in their crystal phases, up to 0.05 Å. These results agree with the earlier made observation that the M-o and M-m and M-f compounds consist of multiple mixed phases, significantly straining their crystal lattices.

SEM analysis confirms the presence of multiple phases in the mixed compounds, in strong contrast to the uniform phase for the pure compounds (Fig. S5†). However, to be able to exactly determine the phases present in the performance-optimized mixed samples, reference peaks of potentially formed phases were compared to the resulting total diffractograms. Fig. 3 shows the measured XRD data plotted above reference delta peaks of possibly present phases, see also Fig. S3.† During the phase-identification, all known XRD patterns containing copper, niobium and or oxygen were considered.^38^ The measured XRD diffractograms show the crystalline monoclinic, orthorhombic and combination of these peaks, around 30°, for M-m, M-o and M-f respectively, together with a number of peaks from other phases. Table 3 lists the ratios of the phases present in the performance-optimized samples, as determined *via* XRD. Fig. 3a shows the phase analysis of sample M-m. The main phase in the sample seems to be h-Nb_2_O_5_, one of the starting materials, which apparently has not reacted for the largest part. This is confirmed by the presence of CuO peaks, which also remains partially unreacted. The figure also shows a fraction of Nb_12_O_29_ at 24°, 25° and 32°, along with an amount of the targeted m-CuNb_2_O_6_ phase. For M-o, the main constituent is the targeted o-CuNb_2_O_6_, but now mixed with a 4.2 mol% fraction of CuNb_3_O_8_, as determined by Rietveld refinement, significantly influencing its electrochemical properties. Fig. 3c presents the XRD phase analysis of the M-f compound. Next to an unreacted amount of 50.9 mol% h-Nb_2_O_5_, and a minor fraction of Nb_12_O_29_, a multiphase of both the monoclinic as well as the orthorhombic copper niobate seems to be present, which is shown by the characteristic triple peak around 30°.

**Table 3:** Molar fractions of phases present in the performance-optimized samples. The table shows the concentrations of the found phases and their summed atomic fractions within the material. The sample fraction was determined *via* Rietveld refinement in the software program Profex^36^

Phase	M-m	M-o	M-f
m-CuNb_2_O_6_ [mol%]	9.0 ± 0.3	—	9.9 ± 1.3
o-CuNb_2_O_6_ [mol%]	—	95.8 ± 0.5	7.9 ± 0.3
CuO [mol%]	40.4 ± 0.9	—	29.9 ± 0.9
h-Nb_2_O_5_ [mol%]	48.9 ± 0.4	—	50.9 ± 1.4
Nb_12_O_29_ [mol%]	1.8 ± 0.1	—	1.4 ± 0.1
CuNb_3_O_8_ [mol%]	—	4.2 ± 0.4	—
Cu [at%]	8.5 ± 0.2	10.9 ± 0.1	7.5 ± 0.2
Nb [at%]	23.8 ± 0.5	22.4 ± 0.1	24.3 ± 0.7
O [at%]	67.7 ± 1.5	66.7 ± 0.4	68.1 ± 1.8

When relating Tables S3† to 3, one can see the total elemental ratios, as determined *via* Rietveld refinement, for M-m and M-f are very near the measured values *via* XRF. For the M-o compound however, a discrepancy is observed between the measured and estimated atomic ratios. Rietveld refinement of the XRD data yields a higher copper concentration than XRF. This may be caused by the presence of an amorphous phase, unaccounted for in Rietveld refinement, or the presence of an elemental concentration gradient between the phases, affecting the total calculated copper concentration in XRD.

**Fig. 4:**
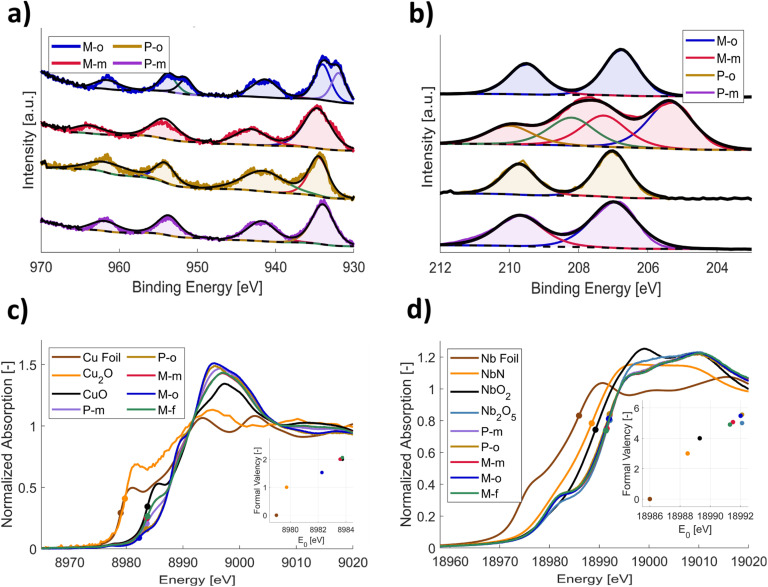
Structural XPS analysis of the pristine sample powders for the characteristic (a) copper 2p and (b) niobium 3d peaks. For peak fitting and position, the area ratio of 1 : 2 for p-orbitals (2p_1/2_ : 2p_3/2_) was taken, while for d-orbitals the ratio of 2 : 3 (3d_3/2_ : 3d_5/2_) was used. The full width at half maximum (FWHM) was set as equal for peaks of the same oxidation state, and positions were taken from Biesinger *et al.*^52^ for Cu and Prudnikava *et al.*^53^ for Nb. (c) and (d) K-edge XANES spectra of (c) copper and (d) niobium, respectively, measured on the phase-pure and performance-optimized samples. The spectra are compared to reference spectra, containing the transition metals in different, known oxidation states. The insets depict linear fitting on the absorption K-edges (*E*_0_) of reference spectra to the samples, in order to obtain their oxidation states.

XPS copper 2p analysis of the P-m, P-o, M-o and M-m compounds shows that all samples exhibit the typical strong 2p_1/2_ and 2p_3/2_ satellite peaks at 942 and 962 eV, respectively, indicative of the Cu^2+^ oxidation state^52^ (Fig. 4a). The Cu 2p_1/2_ main peak is expected at 933.76 ± 0.11 eV for Cu^2+^, whilst the Cu^+^ peak is expected at 932.18 ± 0.12 eV.^52^ Peak fitting of the data, showed that only in M-o the double peak of Cu^+^ phase is present, at a lower binding energy than the Cu 2p_1/2_ main peak. This supports the XRD phase analysis, as CuNb_3_O_8_ is the only phase present in the samples, that contains Cu^+^ (Cu^+^Nb_3_^5+^O_8_^2−^).^54^ Another variation between samples can be observed in the niobium 3d XPS patterns (Fig. 4b). While all samples exhibit the presence of Nb^5+^, by expression of the d_3/2_ peak at 207.60 ± 0.08 eV, M-m also exhibits the Nb^4+^ 3d_3/2_ peak at 206.07 ± 0.19 eV. There seems to be a significant fraction of Nb^4+^ present in the sample, which is in line with the XRD peaks observed in Fig. 3a, as Nb_12_O_29_ is a combination of Nb^4+^ & Nb^5+^ (Nb_2_^4+^Nb_10_^5+^O_29_^2−^).^55^ The Nb^4+^ fraction is still larger than expected however, which could originate from the fact that niobium tends to partly reduce, to form non-stoichiometric oxides near its surface interface, combined with the fact that XPS typically does not penetrate the sample further than 10 nm, possibly giving a false impression of the material's bulk oxidation state.^56^

To further evaluate the bulk valency and confirm the phase analyses of the samples, Cu and Nb K-edge X-ray absorption near edge structure (XANES) was performed (Fig. 4c and d). The dots on the spectra represent the absorption K-edges (*E*_0_) of Cu and Nb in the samples, which have been extracted and subsequently plotted in the insets for their absorption energies. *Via* linear fitting of the samples to the known reference spectra, the formal bulk valency could be determined. For the copper K-edge spectra, all samples, except for M-o, have the same *E*_0_ value as CuO. This aligns with the XPS analysis, *i.e.*, their copper exists purely in the Cu^2+^ oxidation state. For M-o however, the average bulk valency is determined to be lower than 2+, indicating a mixture of Cu^+^ and Cu^2+^, as previously also suggested from the XPS and XRD data. Similarly, for niobium, the P-m, P-o and M-o compounds seem to purely exist in the 5+ oxidation state, whilst the M-m and M-f compounds have lower averaged formal valences. This again, is in line with the XRD and XPS results, indicating the presence of Nb_12_O_29_ in these materials.

### 3.4 Electrochemical performance

**Fig. 5:**
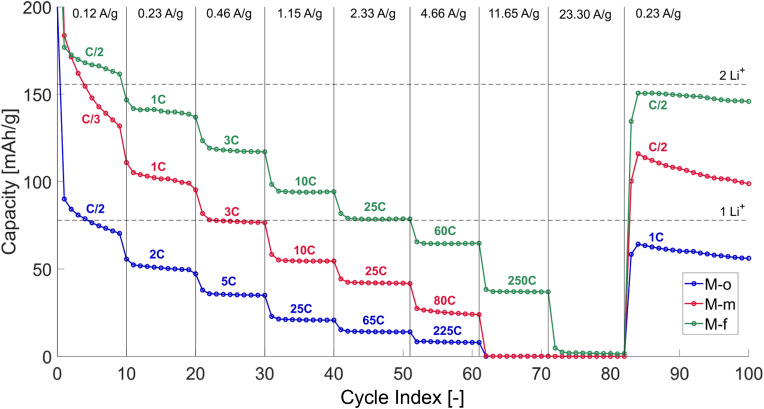
GDC experiments of single half-cells of M-o, M-m and M-f, performed at different current densities. The current density was changed after 10 cycles, and set in the following order: 0.12 A g^−1^ (C/2), 0.23 A g^−1^ (1C), 0.46 A g^−1^ (2C), 1.15 A g^−1^ (5C), 2.33 A g^−1^ (10C), 4.66 A g^−1^ (20C), 11.65 A g^−1^ (50C), 23.30 A g^−1^ (100C) and decreasing back to 0.23 A g^−1^ (1C) for 18 cycles. The experimentally determined C-rates are indicated in the figure and vary from the pre-set C-rates due to variations in the diffusion-resistances in the materials, yielding faster cycling times.

Ultimately, the interest in these novel electrodes lies with their electrochemical performance during battery cycling, *i.e.*, how much the charge rate can be increased and whether the capacity is retained. To this end, GDC of M-o, M-m and M-f half-cells were measured at increasingly higher current densities (Fig. 5). After the loss of a portion of their initial capacity, they remained highly stable at high C-rates. The cycling of the electrodes (M-o, M-m, and M-f) starts around C/2, where they have initial capacities of respectively 200, 280 and 326 mA h g^−1^, whilst forming an SEI layer. Upon doubling the applied current density to 0.23 A g^−1^, the cycling stabilizes at 2C, 1C and 1C with reversible capacities of 51, 101 and 140 mA h g^−1^. Stepwise increase of the current density lowers the reversible capacity slightly, but significantly increases the C-rate. As of such, at an applied current of 2.33 A g^−1^, which corresponds to a theoretical C-rate of 10C, yields discharge times of 54 seconds (65C), 144 seconds (25C) and 150 seconds (25C), but showing reversible capacities of 14, 42 and 79 mA h g^−1^. Upon even further increase of the current densities up to 11.65 A g^−1^, the reversible capacities of M-m and M-o reduces to 0.1 mA h g^−1^. The capacity of M-f however, remains constant at 37 mA h g^−1^, at ultrafast discharge times of 14 seconds (250C). Hence, the material retains up to 25% of its reversible capacity upon a 500 times faster discharge rate. Even more interesting, the cells regain their initial reversible capacity for low cycling rates upon reducing the current density from 23.30 A g^−1^ back to 0.23 A g^−1^. This shows that the cells are highly resilient to large current densities and can operate in a broad range of C-rates, without major degradation. The exceptional electrochemical performance of the mixed-phase anodes is highly reproducible, as demonstrated for the M-f compound in Fig. S6 in the ESI.†

**Fig. 6:**
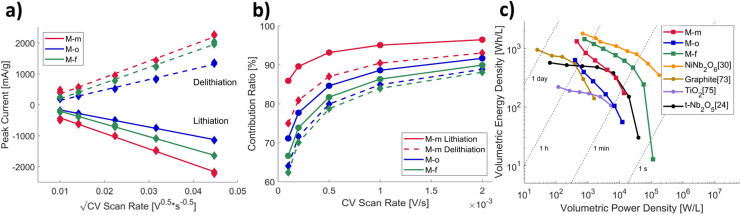
(a) Change in the main peak currents of cycling voltammetry during lithiation and delithiation of the M-m, M-o and M-f electrode materials, at applied sweep rates, as determined *via* Dunn's linearization.^29,62^ (b) Pseudocapacitive contribution ratio to the total electrochemical capacity. (c) Volumetric Ragone plot used to visualize the performance of the anode materials, compared to sources from literature.^23,29,67,68^

To be able to understand the ultrafast charge and discharge processes occurring in the electrode materials, the change in the maximum current through the cell as a function of increasing sweep rate was studied by CV (Fig. 6a). Typically, the peak current grows in size, both during lithiation and delithiation, due to a decrease in the size of the diffusion layer and a higher ionic concentration at the surface.^57,58^

The measured specific surface areas of the materials were determined through nitrogen physisorption. The BET areas were 0.80 ± 0.01 m^2^ g^−1^, 2.29 ± 0.02 m^2^ g^−1^ and 2.47 ± 0.01 m^2^ g^−1^ for M-o, M-m and M-f respectively. The Randles–Sevčík^58–60^ equation is used to calculate the Li^+^-diffusion constants in Table 4. Generally, all of the samples have relatively high Li^+^-diffusion coefficients compared to literature, adding to their resistance to exceptionally high current densities, and allowing for ultra-fast charge and discharging of the electrodes.

**Table 4:** Comparison of lithium-ion diffusion coefficients during lithiation in the performance-optimized samples, as determined *via* the Randles–Ševčík equation,^60^ compared to anode oxide powders from various sources

Material	*D*_Li^+^_ [cm^2^ s^−1^]	Source
M-m	3.8 × 10^−12^	This work
M-o	1.1 × 10^−11^	This work
M-f	1.8 × 10^−12^	This work
NiNb_2_O_6_	1.4 × 10^−12^	29
GaNb_49_O_124_	1.6 × 10^−13^	61
TiNb_10_O_29_	1.8 × 10^−15^	62
LiTi_5_O_24_	8.8 × 10^−16^	63
Nb_14_W_3_O_44_	8.0 × 10^−13^	64
Nb_8_W_9_O_47_	1.0 × 10^−12^	65

To further investigate the charging processes at the electrode, the pseudocapacitive contribution to the total reversible capacity was determined. A high pseudocapacitive contribution is beneficial for next generation batteries^66^ due to its high charging rate capability and its independence of time-consuming intercalation processes. The pseudocapacitive contribution is generated by surface-active effects such as adsorption and redox reactions, which can occur at very short timescales, due to being unrestrained by solid-state diffusion. The pseudocapacitive contribution ratio is derived *via* the Dunn's method for diffusion-controlled insertion processes,^62^ which splits the peak currents measured during CV (Fig. 6a) into a capacitive and a pseudocapacitive contribution, as visualized in Fig. 6b. All measured samples show high pseudocapacitive behaviour, increasing up until 96% for M-m at fast scanning rates during lithiation. This agrees with Fig. 5, showing high flexibility towards faster charge and discharge rates, caused by a high portion of the redox activity occurring at the interphase of the particles.

The increased performance of the mixed-phased anodes in contrast to the phase-pure anodes is surprising. Generally, in battery materials, phase-purity is required to maximize performance-parameters as multiphases typically disrupt the crystal structure. For instance, impurities can limit the measured electrochemical capacity due to partial absence of the wanted redox pairs or negatively impact Li^+^-diffusion in the crystal lattice. As this is clearly not the case for these samples, we can assume a synergistic effect is at play between the various phases. As described in Table 3, M-o is a dual phase, mainly composed of orthorhombic CuNb_2_O_6_, with a 4.2 mol% portion of CuNb_3_O_8_. CuNb_3_O_8_ was described by Huang *et al.*^69^ as a very good Li^+^-conductor (diffusion coefficient of 2.2 × 10^−12^ cm^2^ s^−1^) due to its open crystal structure, with highly stable but poor electrochemical capacity (75 mA h g^−1^ at 1C). The material has been confirmed as a good electronic conductor, due to the presence of Cu^+^ (t_2g_^6^e_g_^4^), with a full 3d electrons shell.^70^ M-m consists of monoclinic CuNb_2_O_6_, CuO, h-Nb_2_O_5_ and Nb_12_O_29_. Here, Cu^2+^ acts as an electron source, due to its (t_2g_^6^e_g_^3^) electronic configuration, increasing the conductivity of the mixture. h-Nb_2_O_5_ is the major constituent in the mixture, as also confirmed by its cyclic voltammogram similar to M-m, as shown in the ESI.† h-Nb_2_O_5_ has a high initial capacity that quickly fades due to the low electronic conductivity of Nb^5+^ (t_2g_^0^e_g_^0^), in literature solved by copper doping.^30,31,71^ Nb_12_O_29_ has a ReO_3_ crystal structure and consists of a combination of 2 Nb^4+^ (t_2g_^1^e_g_^0^) and 10 Nb^5+^ (t_2g_^0^e_g_^0^) ions, increasing the amount of free electrons in the system. Overall, Nb_12_O_29_ has better electronic conductivity than h-Nb_2_O_5_, but lower capacity due to the partial loss of Nb^5+^-ions.^72,73^ Lastly, the same phases are present in M-f as in M-m, albeit at different ratios, while containing a 7.9 mol% portion of orthorhombic CuNb_2_O_6_. It is likely that the ratio of these phases determines the overall anode performance, and might be further optimized.

The M-f compound thus contains both crystal phases of CuNb_2_O_6_, of which the orthorhombic system is the more symmetrical one. Higher crystal symmetry is beneficial for Li^+^-diffusion, while lower symmetry may create localized electronic states, possibly narrowing the effective band gap of the material, adding to its electronic conductivity. M-f has the best performance in terms of reversible capacity and rate stability. This is likely a result of synergy between its constituent phases, where the copper-rich phases deliver electrons needed for conductivity and stability, and the niobium-rich phases are responsible for the redox activity, and hence the reversible capacity.

The volumetric Ragone plot (Fig. 6c), compares the volumetric energy and power densities of M-o, M-m, and M-f obtained from Fig. 5 to experimental values from literature. In terms of energy density, all three samples have relatively high energy densities at low charging rates. Especially M-f, retains its competitive energy density at higher discharge rates, similar to the values found for NiNb_2_O_6_. At low discharge rates (1C), the volumetric energy densities of M-o, M-m and M-f are 820 W h L^−1^, 908 W h L^−1^ and 1640 W h L^−1^ respectively, which strongly competes with the current state-of-the-art Li-ion battery anodes, graphite (400 W h L^−1^) and LTO (280 W h L^−1^).^67,74^ At a similar energy density, M-f an almost 70 times higher power density (27 000 W L^−1^). This is, compared to literature sources a highly competitive result, and omits the usage of the increasingly more critical nickel metal.

## 4 Conclusions

Phase-pure orthorhombic (P-o) and monoclinic (P-m) copper niobate samples were synthesized *via* a facile solid-state reaction method, where the applied annealing temperature determines its phase. The materials were shown to have low (45 and 25 mA h g^−1^), but stable (99.51%, 98.37%) reversible capacities (at a current of 233 mA g^−1^), exhibiting a host crystal structure with large open channels wherein lithium can reversibly intercalate. To increase the reversible capacity and stability, the materials synthesis was steered towards the formation of multiple phases. A novel electrode material (M-f) consisting of five different phases displays the highest performance. It was determined that copper-rich phases are responsible for the high electronic conductivity and hence cycle stability and niobium-rich phases are responsible for the high redox capacity and superior cycling rates. This is in contrast with earlier observed results concerning other columbite anode materials, where the main phase is the most electrochemically active. M-f exhibited a high electrochemical capacity of 167 mA h g^−1^ (*I* = 233 mA g^−1^), at a capacity retention of 99.81% after 100 cycles. Moreover, the anodes proved to be resilient upon subjection to very large current densities (up to 23 A g^−1^), and exhibiting high reversible capacity at fast discharge times (95 mA h g^−1^ at 10C, 65 mA h g^−1^ at 60C and 37 mA h g^−1^ at 250C). These results indicate that the material retains up to 25% of its reversible capacity with an almost 500 times faster discharge rate. Moreover, after being subjected to these high current densities, the material was observed to recover its initial capacity upon lowering the current density back to 1C, indicating the high stability of the materials. These high C-rate performances were found to be caused by a high Li^+^-diffusion constant (1.8 × 10^−12^ cm^2^ s^−1^) and pseudocapacitive behaviour (up to 90%). As compared to graphite, the anodes demonstrate a nearly 70 times greater power density (27 000 W L^−1^) for the same energy density (470 W h L^−1^) with a discharge duration of merely 62 seconds. Ultimately, copper niobate has been shown to be a promising alternative to current lithium-ion battery anodes, exhibiting fast intercalation kinetics and high capacities at elevated C-rates, which is useful for both the EV industry as well as the development of responsive, rapid charging energy storage systems, while consisting fully of non-toxic and abundant elements.

## Data availability

The raw data will be available at the 4TU data repository: https://data.4tu.nl/.

## Author contributions

B. M. Jager: writing – review & editing, writing – original draft, visualization, validation, methodology, investigation, formal analysis, conceptualization. L. Kortekaas: writing – review & editing. J. E. ten Elshof: writing – review & editing, supervision. J. W. G. Bos: writing – review & editing, supervision. M. Tromp: writing – review & editing, supervision, conceptualization, resources. M. Huijben: writing – review & editing, writing – original draft, supervision, resources, funding acquisition, conceptualization.

## Conflicts of interest

The author(s) declare(s) that there is no conflict of interest regarding the publication of this article.

## Supplementary Material

TA-013-D4TA07548J-s001
