# Supplementary material for: Mixed-phase enabled high-rate copper niobate anodes for lithium-ion batteries
Source: J Mater Chem A Mater. 2025 Jan 8;13(7):5130–42. doi: 10.1039/d4ta07548j (PMC11737045; doi:10.1039/d4ta07548j)
Supplement: TA-013-D4TA07548J-s001 [file TA-013-D4TA07548J-s001.pdf]

Supplementary Information (SI) for Journal of Materials Chemistry A.
This journal is © The Royal Society of Chemistry 2025

Mixed-Phase Enabled High-Rate Copper Niobate Anodes for Lithium-Ion Batteries

B. Maarten Jager[a], Luuk Kortekaas[a], Johan E. ten Elshof[b], Jan-Willem G. Bos[c], Moniek Tromp[a], and Mark Huijben[b*]

[a]Zernike Institute for Advanced Materials, University of Groningen, 9747 AG Groningen, Netherlands; E-mail: moniek.tromp@rug.nl
[b]MESA+ Institute for Nanotechnology, University of Twente, 7500 AE Enschede, Netherlands; E-mail: m.huijben@utwente.nl
[c]EaStCHEM School of Chemistry, University of St Andrews, KY16 9ST St Andrews, UK; E-mail: j.w.g.bos@st-andrews.ac.uk

Supplementary Materials

Table S1: Comparison of lattice parameters of various columbite materials.

**Component**	**Crystal**	**a** [Å]	**b** [Å]	**c** [Å]	α [°]	β [°]	γ [°]	**V** [Å^3]	**Source**
$CaNb_2O_6$	Orthorhombic	14.92	5.75	5.20	90	90	90	446	[1]
$CrNb_2O_6$	Tetragonal	4.72	4.72	3.01	90	90	90	67	[2]
$CuNb_2O_6$	Monoclinic	14.17	5.76	5.01	90	90	92	409	[3]
$CuNb_2O_6$	Orthorhombic	14.10	5.61	5.12	90	90	90	405	[3]
$FeNb_2O_6$	Orthorhombic	14.25	5.73	5.05	90	90	90	412	[2]
$LiNb_3O_8$	Monoclinic	15.26	7.46	5.03	90	90	107.34	547	[1]
$MgNb_2O_6$	Orthorhombic	14.18	5.70	5.03	90	90	90	407	[1]
$MnNb_2O_6$	Orthorhombic	14.42	5.76	5.08	90	90	90	422	[3]
$NiNb_2O_6$	Orthorhombic	14.02	5.68	5.02	90	90	90	400	[4]
$ZnNb_2O_6$	Orthorhombic	14.21	5.72	5.04	90	90	90	410	[3]

Table S2: Summary of columbite-based anode performance for lithium-ion batteries as reported in literature. The table lists all columbite materials currently studied for LIBs, showing the achieved reversible capacities (C_p) per cycle count (#), at specified current densities (j). Hyphens (-) denote missing specific data in the cited article, while question marks (?) indicate the article does not specify the current density used.

**Component**	**Crystal**	$\mathbf{C_{p,high}}$ $[\mathbf{mAh \cdot g^{-1}}]$ (#)	**j** $[\mathbf{mA \cdot g^{-1}}]$	$\mathbf{C_{p,low}}$ $[\mathbf{mAh \cdot g^{-1}}]$ (#)	**j** $[\mathbf{mA \cdot g^{-1}}]$	**Source**
$CrNb_2O_6$	Tetragonal	16 (10)	?	-	-	[2]
$CoNb_2O_6$	Orthorhombic	300 (500)	200	50	500	[5]
$CuNb_2O_6$	Monoclinic	256 (20)	38 (0.1C)	131 (20)	3,800 (10C)	[6]
$CuNb_2O_6$	Orthorhombic	350 (32)	50	155 (28)	5,000	[7]
$FeNb_2O_6$	Orthorhombic	35 (10)	?	-	-	[2]
$LiNb_3O_8$	Monoclinic	150 (50)	(0.01C)	-	-	[1]
$LiNb_3O_8$	Monoclinic	200 (50)	(0.05C)	150 (50)	(0.1C)	[8]
$MnNb_2O_6$	Orthorhombic	275 (20)	38 (0.1C)	20 (20)	3,800 (10C)	[6]
$NiNb_2O_6$	Orthorhombic	244 (300)	(1C)	50 (20,000)	(100C)	[4]
$NiNb_2O_6$	Orthorhombic	250 (100)	50 (5C)	100 (90)	2,400 (10C)	[9]
$ZnNb_2O_6$	Orthorhombic	300 (20)	38 (0.1C)	25 (20)	3,800 (10C)	[6]

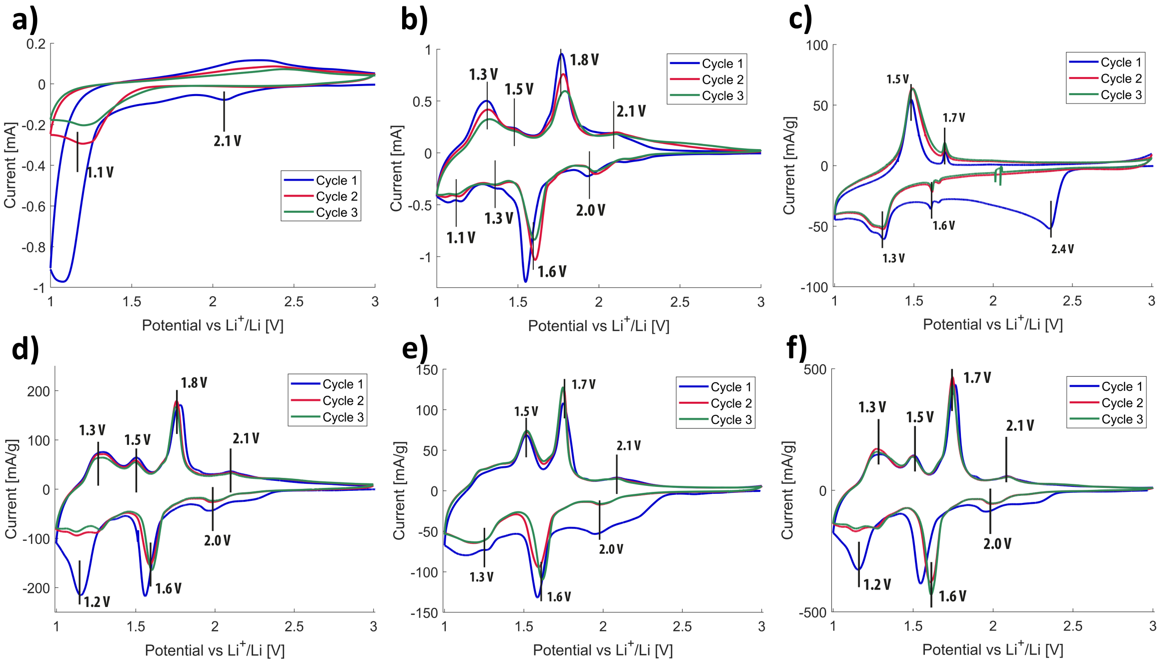

Figure S1: Cyclic voltammograms measured at a scanning rate of 0.1 mV/s, between 3.0-1.0 V, which was repeated for 2 additional cycles. Potentials of specific peaks are indicated. a) CuO, b) h−Nb_2O_5, c) P-o, d) M-m, e) M-o and f) M-f.

Figure S1 shows CV data of pristine reactant powders CuO and h−Nb_2O_5, which are used to explain the electrochemical processes occurring during charging and discharging of the copper niobate anodes. Figure S1a shows the obtained CV data of CuO. In the first cathodic scan, a weak broad peak at 2.1 V and a strong peak at 1.1 V can be seen. These correspond to irreversible multistep electrochemical reactions, with at 2.1V[10]:

$$CuO + x\ Li^+ + x\ e^- \longrightarrow Li_xCuO \qquad (S1)$$

Where the copper oxide reacts with lithium to form a solid solution phase. The sharp peak at 1.1 V is associated with structural destruction and subsequent formation of Cu_2O[10, 11]:

$$2\,CuO + 2\,Li^+ + 2\,e^- \longrightarrow Cu_2O + Li_2O \quad \textit{or} \quad 2\,Li_xCuO \longrightarrow Cu_2O + x\ Li_2O \qquad (S2)$$

Here, Cu(II) is reduced to Cu(I), under the formation of an amorphous layer of Li_2O, following the growth of the solid electrolyte interface (SEI). The formation of the SEI layer limits the electrochemical activity of the electrode. In subsequent cycles, at 1.2/2.3 V partial re-oxidation of Cu_2O into Li_xCuO occurs, resulting in a small amount of working capacity[10, 11, 12]:

$$2\,Li_xCuO \longleftrightarrow Cu_2O + x\ Li_2O \qquad (S3)$$

The repeated structural destruction and transformation of Li_xCuO into CuO and amorphous Li_2O, progresses the formation of the SEI layer, which weakens the connection to the catalytic sites, leading to poor cycleability and rate performance[12].

In figure S1b, the cyclic voltammogram of the $H-Nb_2O_5$ precursor is shown. The electrochemical behaviour is relatively stable, with some loss of capacity and slight peak movement after the first cycle, due to the SEI layer formation. The most prominent is the peak located at 1.6/1.8 V, which is linked to the reversible reduction of Nb^{5+} to Nb^{4+}, due to lithium intercalation[13]:

$$Nb_2O_5 + 2\,Li^+ + 2\,e^- \longleftrightarrow Li_2Nb_2O_5 \qquad (S4)$$

The redox couples present at 1.3/1.5 and 1.1/1.3 V are likely caused by the further partial reduction of Nb^{4+} to Nb^{3+}, possibly occurring in two stages due to the different chemical environment of the ions[13]:

$$Li_2Nb_2O_5 + x\ Li^+ + x\ e^- \longleftrightarrow Li_{2+x}Nb_2O_5 \qquad (S5)$$

The peaks present at 2.0/2.1 are reported in various sources of literature for $H-Nb_2O_5$, but no explanation of a possible redox couple is given however[14, 15, 13, 16].

Figure S1c shows CV data of P-o, which has a similar shape as the CV data of P-m as shown in the main manuscript, but different peak ratios. This means that the same electrochemical processes take place in these samples, but not every reaction contributes equally to the total capacity. Figure S1d-f present electrochemical data obtained by CV, for the samples M-m, M-o and M-f. The CV data of M-m seems to be a combination of the CV data of $h-Nb_2O_5$ and P-m, due to the increased presence of the peak at 1.1/1.3 V, reduction of the peaks at 1.3/1.5 V, and the formation of the SEI layer at 2.3 V. This is, to a lesser degree, also the case for M-o, where the data at 2.4 and 1.5 - 1.0 V highly resembles the P-o data, but the peak intensities of 1.6/1.7 V and 1.3/1.5 V seem

to have been flipped to the configuration of Nb_2O_5. M-f shows very similar behaviour to M-m and h$-Nb_2O_5$, meaning the largest part of the electrochemistry occurs due to this phase and is mostly only stabilized by CuO and the other phases.

Table S3: Sample-averaged atomic fractions of P-m, P-o, M-o, M-m and M-f, as determined by XRF and compared to the theoretical atomic ratios for pure copper niobate.

**Atom**	**$CuNb_2O_6$**	**P-m**	**P-o**	**M-m**	**M-o**	**M-f**
Cu [at%]	11.1	11.4 ± 0.2	11.2 ± 0.1	7.7 ± 0.2	7.7 ± 0.2	7.4 ± 0.2
Nb [at%]	22.2	22.1 ± 0.2	22.2 ± 0.2	23.1 ± 0.2	23.1 ± 0.2	24.4 ± 0.2
O [at%]	66.7	66.5 ± 0.8	66.6 ± 0.9	69.2 ± 1.8	69.2 ± 1.5	68.3 ± 1.8

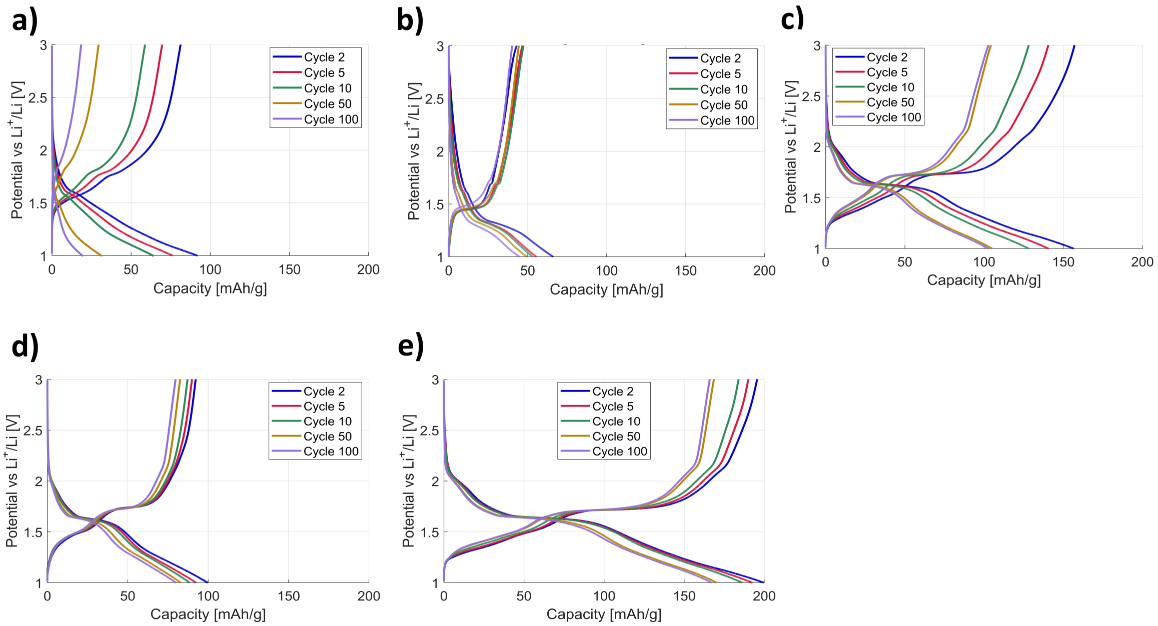

Figure S2: Charge/Discharge profiles at selected cycles as measured during GDC at 1C, 233 mA/g for 100 cycles between 3 - 1 V vs Li^+/Li for a) P-m, b) P-o, c) M-m, d) M-o and e) M-f.

Table S4: Calculated lattice parameters (a, b and c), lattice volumes (V) and crystallite sizes (L) of selected copper niobate samples via Rietveld refinement, compared to reference values. For M-f the lattice parameters of both copper niobate phases are given.

**Sample**	**a [Å]**	**b [Å]**	**c [Å]**	**β [°]**	**V [Å^3]**	**L [nm]**
m−$CuNb_2O_6$[3]	14.173	5.762	5.006	91.67	408.6	-
o−$CuNb_2O_6$[3]	14.097	5.613	5.123	90	405.4	-
P-m	14.182 ± 0.003	5.762 ± 0.001	5.008 ± 0.001	91.72 ± 0.01	409.0 ± 0.1	102 ± 26
P-o	14.126 ± 0.006	5.612 ± 0.002	5.127 ± 0.002	90	406.4 ± 0.2	46 ± 1
M-m	14.136 ± 0.003	5.733 ± 0.003	5.044 ± 0.002	91.65 ± 0.02	408.6 ± 0.2	46 ± 5
M-o	14.059 ± 0.001	5.559 ± 0.001	5.108 ± 0.001	90	399.2 ± 0.1	38 ± 1
M-f (m)	14.216 ± 0.007	5.771 ± 0.003	4.995 ± 0.002	91.46 ± 0.02	409.7 ± 0.2	215 ± 65
M-f (o)	14.135 ± 0.005	5.618 ± 0.002	5.130 ± 0.002	90	407.4 ± 0.1	70 ± 3

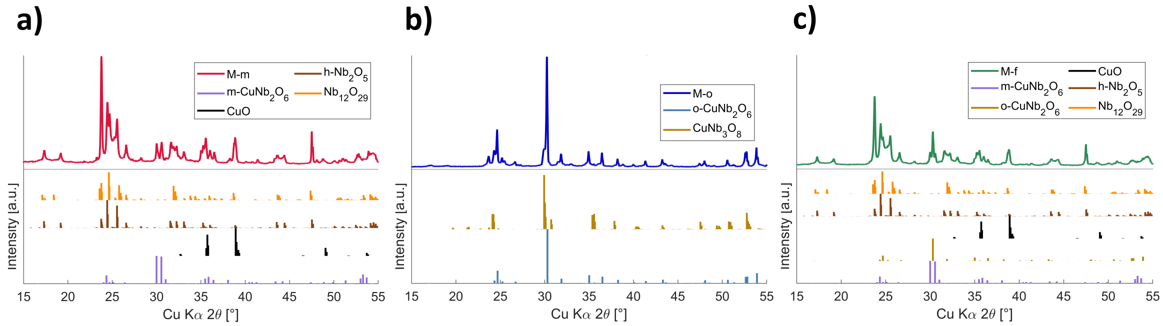

Figure S3: XRD phase analyses of a) M-m, b) M-o, and c) M-f. The XRD diffractograms are plotted above the constituent Rietveld-simulated data to visualize the presence of all peaks.

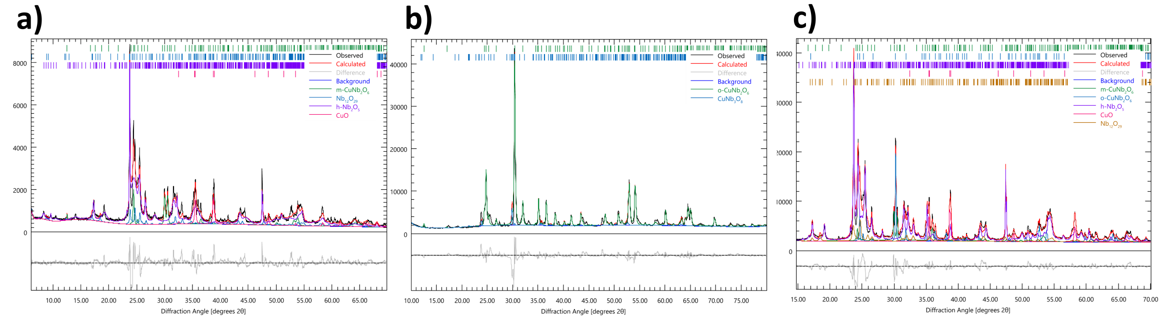

Figure S4: XRD phase analyses of a) M-m, b) M-o, and c) M-f. Rietveld analysis performed in the software program Profex[17] shows a comparison between the measured and the calculated diffractogram by combinations of the reference peaks.

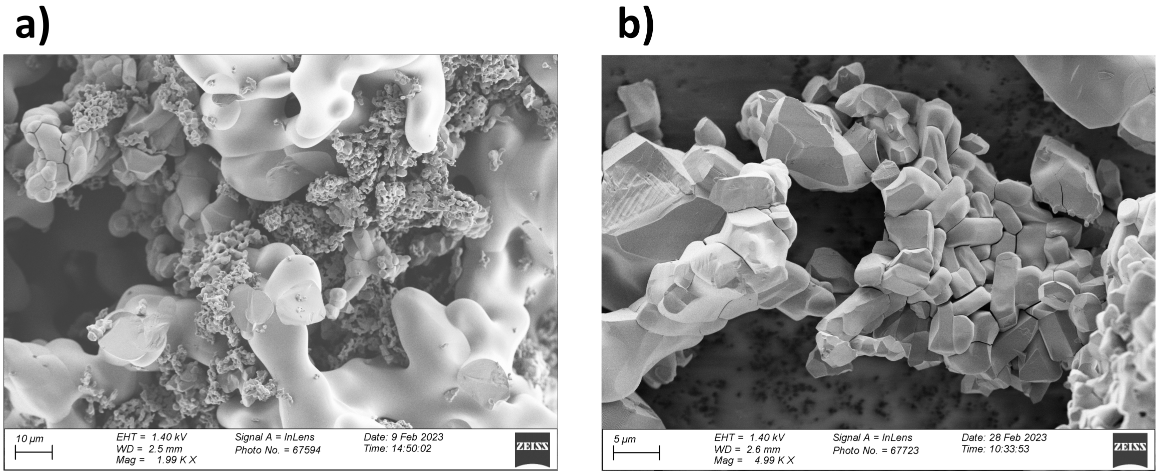

Figure S5: SEM images of the microstructures of the M-o and P-o powder samples. It can be clearly observed that the M-o consists of multiple phases, while the P-o is uniform in its composition.

Figure S5a and b show SEM images of both the mixed (M-o) and phase-pure (P-o) orthorhombic $CuNb_2O_6$ samples. In the former it is clearly visible that multiple phases exist in a mixed state in the powder, whereas for the latter, only one phase is visible, confirming the multi-phase and single-phase nature of both powders.

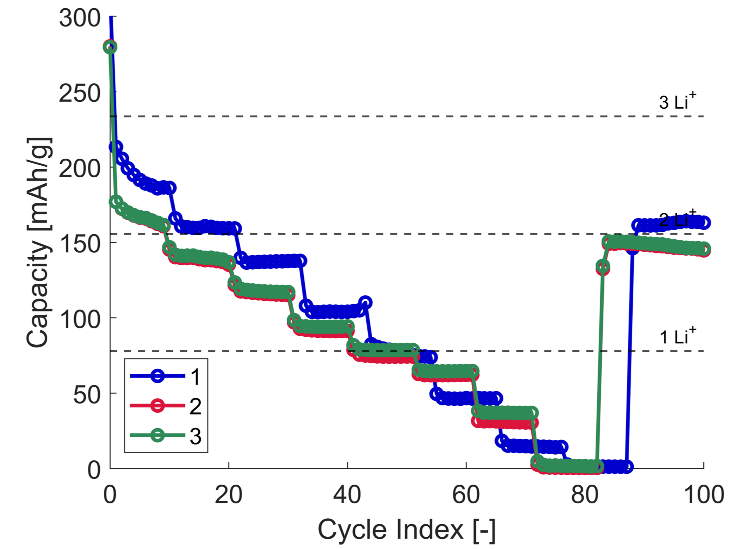

Figure S6: C-rate variation data for three half cells containing electrodes of M-f compound to demonstrate reproducibility.

References

[1] M.A. Reddy and U.V. Varadaraju. "Lithium Insertion into Niobates with Columbite-Type Structure: Interplay between Structure-Composition and Crystallite Size". In: *J. Phys. Chem. C* 115 (2011). DOI: 10.1021/jp206505y.

[2] P.B. Samarasingha, C.I. Thomas, and H. Fjellvåg. "Investigation of Li^+ insertion in columbite structured $FeNb_2O_6$ and rutile structured $CrNb_2O_6$ materials". In: *Electrochim. Acta* 153 (2015). DOI: 10.1016/j.electacta.2014.12.004.

[3] S. Grazulis et al. "Crystallography Open Database - an open-access collection of crystal structures". In: *J. Appl. Crystallogr.* 42 (2009). DOI: 10.1107/S0021889809016690.

[4] R. Xia et al. "Nickel Niobate Anodes for High Rate Lithium-Ion Batteries". In: *Adv. Energy Mater.* 12 (2022). DOI: 10.1002/aenm.202102972.

[5] Y. de Luna et al. "Highly Stable Free-standing Cobalt Niobate with Orthorhombic Structure as Anode Material for Li-ion Batteries". In: *ChemElectroChem* 11 (2024). DOI: 10.1002/celc.202300627.

[6] S. Lee et al. "Copper, zinc, and manganese niobates: Structural characteristics of Li^+ storage properties, and working mechanisms". In: *Inorg. Chem. Front.* 7 (2020). DOI: 10.1039/d0qi00475h.

[7] M. Li et al. "High-rate capability of columbite $CuNb_2O_6$ anode materials for lithium-ion batteries". In: *Mater. Lett.* 284 (2021). DOI: 10.1016/j.matlet.2020.128915.

[8] Z. Jian et al. "$LiNb_3O_8$ as a novel anode material for lithium-ion batteries". In: *Electrochem. Commun.* 13 (2011). DOI: 10.1016/j.elecom.2011.07.018.

[9] Y. de Luna and N. Bensalah. "Mechanochemical Synthesis of Orthorhombic Nickel Niobate as a Robust and Fast Charging Anode Material for Lithium-Ion Batteries". In: *ACS Appl. Energy Mater.* 5 (2022). DOI: 10.1021/acsaem.2c00935.

[10] R. Sahay et al. "High Aspect Ratio Electrospun CuO Nanofibers as Anode Material for Lithium-Ion Batteries with Superior Cycleability". In: *J. Phys. Chem. C* 116 (2012). DOI: 10.1021/jp3053949.

[11] D. Yin et al. "CuO Nanorod Arrays Formed Directly on Cu Foil from MOFs as Superior Binder-Free Anode Material for Lithium-Ion Batteries". In: *ACS Energy Lett.* 2 (2017). DOI: 10.1021/acsenergylett.7b00215.

[12] C. Wang et al. "Morphology-Dependent Performance of CuO Anodes Via Facile and Controllable Synthesis for Lithium-Ion Batteries". In: *ACS Appl. Mater. Interf.* 6 (2014). DOI: 10.1021/am405061c.

[13] T. Li et al. "A niobium oxide with a shear structure and planar defects for high-power lithium ion batteries". In: *Energy Environ. Sci.* 15 (2022). DOI: 10.1039/d1ee02664j.

[14] Z. Song et al. "Ultrafast and Stable Li-(De)intercalation in a Large Single Crystal $H-Nb_2O_5$ Anode via Optimization the Homogeneity of Electron and Ion Transport". In: *Adv. Mater.* 32 (2020). DOI: 10.1002/adma.202001001.

[15] S. Huang et al. “Single crystal $H{-}Nb_2O_5$ growing along the [001] crystal direction for ultrafast lithium storage”. In: *J. Mater. Chem. A* 11 (2023). DOI: 10.1039/d2ta07935f.

[16] D. Cao et al. “$H{-}Nb_2O_5$ wired by tetragonal tungsten bronze related domains as high-rate anode for Li-ion batteries”. In: *Energy Storage Mater.* 11 (2018). DOI: 10.1016/j.ensm.2017.10.005.

[17] N. Doebelin and R. Kleeberg. “Profex: a graphical user interface for the Rietveld refinement program BGMN”. In: *J. Appl. Crystal.* 48 (2015). DOI: 10.1107/S1600576715014685.
